# What’s leak got to do with it? Association of mask leak and positive airway pressure adherence from the homepap study

**DOI:** 10.1007/s44470-026-00046-2

**Published:** 2026-04-10

**Authors:** Noah D. Andrews, Jad El Ahdab, Maeve Pascoe, Lu Wang, James Bena, Dennis Auckley, Ruth Benca, Martha E. Billings, Vishesh Kapur, Phyllis C. Zee, Susan Redline, Nancy Foldvary-Schaefer

**Affiliations:** 1 Cleveland Clinic Sleep Disorders Center, Cleveland, OH USA; 2 Cleveland Clinic Quantitative Health Sciences, Cleveland, OH USA; 3 Metro Health Medical Center, Case Western Reserve University School of Medicine, Cleveland, OH USA; 4 Department of Psychiatry and Behavioral Medicine, Wake Forest University School of Medicine, Winston-Salem, NC USA; 5 Harborview Medical Center, University of Washington, Seattle, WA USA; 6 Feinberg School of Medicine, Northwestern University, Chicago, IL USA; 7 Brigham and Women’s Hospital, Harvard Medical School, Boston, MA USA; 8 Cleveland Clinic Sleep Disorders Center, 9500 Euclid Avenue, S73, Cleveland, OH 44195 USA

**Keywords:** Positive airway pressure (PAP) therapy, HomePAP, Obstructive sleep apnea (OSA), PAP adherence, Mask leak, Unintentional leak, Real leak, Average leak, HomePAP trial

## Abstract

**Background:**

Positive airway pressure (PAP) therapy is the gold standard treatment for obstructive sleep apnea (OSA), yet adherence remains suboptimal. Mask leak is a common barrier, but current leak metrics don’t distinguish intentional from unintentional leak. We evaluated a novel “Real Leak” measure and compared its relationship to adherence with conventional leak metrics.

**Methods:**

We conducted a secondary analysis of the HomePAP trial, which randomized adults at high risk for OSA to home sleep apnea testing or in-laboratory polysomnography, followed by PAP initiation. Real Leak was calculated by subtracting mask-specific intentional leak from device-reported Average Leak, essentially representing unintentional leak over 1 month. PAP adherence was defined as *≥* 4 h/night on ≥ 70% of nights at 1 and 3 months. Pearson correlations and multivariable linear regression adjusted for age, sex, BMI, race, education, Epworth Sleepiness Scale, and apnea-hypopnea index.

**Results:**

Data were available for 139 and 124 participants at 1 and 3 months, respectively. At 3 months, adherence was 46.2%. Real Leak and Average Leak were highly correlated and inversely associated with adherence (rho[95%CI]: 1-month 0.73 [0.61,0.81], 3-month 0.90 [0.85,0.93], *p* < 0.001) at both timepoints. Each 1 L/min increase in Real Leak corresponded to a 0.72% decrease in adherence days, compared to 0.55% in Average Leak (*p* = 0.008/0.018) (7.2% and 5.5% per 10 L/min of change, respectively, *p* = 0.008/0.018). Race and AHI (*p* = 0.02/*p* < 0.001, respectively) were independent adherence predictors.

**Conclusions:**

Both Real Leak and Average Leak negatively predicted PAP adherence, with no significant performance difference. Nonetheless, standardizing leak terminology and incorporating intentional leak adjustments may improve clarity and decision-making.

**Brief summary:**

**Current knowledge/study rationale:**

Mask leak is a common barrier to Positive airway pressure (PAP) adherence, but existing leak metrics don’t distinguish intentional from unintentional leak, limiting their clinical utility. The American Academy of Sleep Medicine emphasizes minimizing leak during PAP titration but doesn’t provide clear guidance on which leak metric to prioritize. We proposed a novel “Real Leak” measure as a potentially more clinically meaningful indicator of leak-related adherence barriers.

**Study impact:**

In the HomePAP trial, Real Leak and Average Leak were highly correlated and similarly predicted reduced adherence, with no significant performance difference. We propose standardizing leak definitions for clinical practice and research and suggest that Real Leak - essentially unintentional leak over 1 month - is a conceptually clearer and more meaningful metric.

## Introduction

Positive airway pressure (PAP) therapy is considered the gold standard treatment for moderate-to-severe obstructive sleep apnea for decades, yet patient adherence continues to be a significant challenge [1]. Despite advancements in PAP technology and treatment modalities, a substantial proportion of patients struggle to meet the recommended usage guidelines. Factors such as mask fit, comfort, and technical issues like air leak can undermine the effectiveness of PAP therapy, ultimately reducing adherence and therapeutic benefit. For instance, studies suggest that up to 66% of PAP users are non-adherent based on current criteria [2]. Adherence criteria is based on the “4 and 70” rule, where average use of PAP therapy *≥* 4 h for at least 70% of nights constitutes acceptable adherence [3]. 

PAP titration procedures (optimal titration pressure), technology (i.e. interface/mask, heated humidification), and psychosocial factors (coping skills, optimism about treatment, self-reliance, beliefs on claustrophobia), education about therapy, and interventions that address patient complaints, as well as age and body mass index (BMI) affect adherence [4, 5]. Troubleshooting mask complaints and device settings is often necessary to address leaks, comfort, and mask fit, which contribute to poor adherence [6].

American Academy of Sleep Medicine (AASM) guidelines for PAP titration emphasize the importance of minimizing mask leak which takes into consideration intentional leak defined as the necessary leak a specific mask should have at a specific pressure to avoid CO_2_ saturation in the mask and elicit hypercapnia [7]. However, specific recommendations for measuring and adjusting for unintentional leak are not provided [8]. A 2017 review of 21 studies addressing mask leak and PAP adherence drew no firm conclusions leaving clinicians to wonder “What’s leak got to do with it?” [9]. According to current guidelines for PAP titration, mask leak should be assessed hourly and addressed if exceeding 20 L/hour [10]. But which leak are technologists supposed to pay attention to? Most PAP machines record at least 4 different types of leak, and aside from the easy decision to exclude “time at large leak” during a sleep study, there are still 3 different types of leak to choose from: Average leak, 90% leak, and Average large leak. The current therapeutic standard which uses Average Leak or twice the expected leak is associated with poor adherence [11]. We hypothesize that the Real Leak measure, which adjusts for unintentional leak by taking into consideration intentional leak as a contributing factor to the total leak, is more closely associated with PAP therapy adherence than traditional leak measures using the HomePAP trial dataset. Additionally, we aim to improve clarity regarding leak-related barriers to PAP adherence and inform clinical practices to enhance patient outcomes.

## Methods

### Sample population

Data were collected in the HomePAP trial, which randomized participants with high probability of moderate-to-severe OSA to home sleep apnea testing (HSAT) or in-laboratory polysomnography (PSG) [12]. The trial collected data from 7 AASM-accredited academic sleep centers across the United States in 5 cities; Case Western Reserve University affiliates: University Hospitals (19 participants, 12.7%), MetroHealth Medical Center (25 participants, 16.7%), Cleveland Clinic (38 participants, 25.3%) in Cleveland, OH; Northwestern University (21 participants, 14.0%) in Chicago, IL; University of Wisconsin (8 participants, 5.3%) in Madison, WI; University of Minnesota (10 participants, 6.7%) in Minneapolis, MN; and University of Washington (29 participants, 19.3%) in Seattle, WA; recruitment was not equal across centers. High probability of moderate-to-severe OSA was defined as an “adjusted neck circumference” of 43 cm or greater, calculated as the patient’s measured neck circumference plus 3 cm for habitual snoring, 4 cm for presence of hypertension, and 3 cm for witnessed apneas/choking/gasping on most nights; an Epworth Sleepiness Scale (ESS) score of *≥* 12 was also required to be eligible. Patients were excluded if they had a history of heart failure, alcohol use defined as more than 5 alcoholic drinks per day, chronic narcotic use, chronic lung disease, severe restless legs syndrome, severe insomnia, narcolepsy, or any psychiatric disorders other than mild depression.

### Study procedures

Participants who met study eligibility criteria completed baseline demographic surveys and received either HSAT or PSG; those with an apnea-hypopnea index (AHI) *≥* 15 were randomized to auto PAP (APAP) titration at home or in-laboratory PAP titration. After successfully completing the titration phase of the study, optimal titration pressure (derived from either APAP download or in-lab titration) and mask-specific intentional leak values, as provided by the manufacturer for each mask type and pressure setting, were recorded. Mask type distribution in our sample was 62.6% nasal, 18.5% nasal pillows, and 16.9% full face masks. Participants were educated by a study technologist about PAP treatment; total time (in minutes) spent educating was recorded. All Participants were provided with REMStar Auto PAP devices and data were centrally stored in Respironics’ Encore Pro (Ver. 1.8.49) software.

During the pretreatment period, participant optimism about PAP treatment was assessed on a 7-point Likert scale. PAP optimism reflects participants’ attitude towards PAP treatment before initiating therapy, with a “1” representing “extremely optimistic” and a “7” representing “extremely pessimistic”.

PAP adherence was evaluated at 1 and 3-months post-titration and defined as *≥* 4 or more hours on *≥* 70% of nights [13]. Data collected at the 1- and 3-month points reflected usage from the prior 30 days. Participants were encouraged to contact study staff at any point to troubleshoot concerns. Interventions related to mask, device, pressure setting, and other basic troubleshooting were addressed through telephone contacts and in-person visits.

Average PAP use was recorded as the mean hours of PAP use over each recording time. To generate a meaningful representation of leak, we calculated a new measure called “Real Leak.” Real Leak was derived from “Average Leak,” the average leak recorded for the usage period over 1 month and evaluates unintentional leak by subtracting the intentional leak from the total leak. Essentially, Real Leak is unintentional leak over a period of 1 month. Intentional leak is calculated by PAP mask manufactures as the necessary leak a specific mask should have at a specific pressure to avoid CO_2_ saturation in the mask and elicit hypercapnia. Average Leak, as the basis for the Real Leak calculation, was chosen for its general acceptance in clinical practice and the propensity for error of other available options, such as “Average Max Leak”, the daily maximum leak averaged over the usage period, and “Average Large Leak”, time spent with leak > 2 times than estimated expected leak [11]. 

### Statistical analysis

Data are presented as mean ± standard deviation or median [25th, 75th percentiles] for continuous variables based on distribution and N (%) for categorical variables. Kurtosis and Skew were tested to assess the distributions of Real Leak versus Average Leak variables. Because Real Leak is derived from Average Leak by subtracting participant-specific intentional leak values (based on mask type and pressure), these measures are expected to be strongly correlated and are not independent. Pearson’s correlation assessed the relationship between PAP adherence and leak measures. Linear regression evaluated the association between adherence and leak adjusted for age, BMI, gender, race, education, ESS at baseline, and AHI. All statistical tests assumed a significance level of 0.5 and both p-values and 95% confidence intervals (CI) are reported. SAS version 9.3 (The SAS Institute Cary, NC) was used to perform all analyses.

## Results

### Sample characteristics

Of the 150 participants, data were available for 139 and 124 at 1 and 3 months, respectively. Data card failure occurred in 11 and 24 cases at 1 and 3 months and 2 participants had no reported use at 3 months.

Sample characteristics are shown in Table 1. Participants had mean age of 48.4 ± 12 years and were predominantly white, male, and overweight or obese. The median AHI was indicative of severe OSA (AHI median [IQR] 36.8 [23.3, 63.5]). Nearly three-quarters were moderately to extremely optimistic about PAP therapy. Participants were educated on PAP therapy for a total time of 44.8 ± 18.8 min and 41.3% had one or more interventions to address PAP challenges.

**Table 1:** Sample characteristics (*N* = 150)

Demographics	
Age, yr	48.4 ± 12.0
Gender, male	98 (65.3)
Race/Ethnicity	
White Black Other	103 (68.7) 27 (18.0) 20 (13.3)
BMI, kg/m^2^	36.3 [31.6, 43.4]
ESS baseline	14.0 [12.0, 16.0]
*Diagnostic PSG & PAP Data*	
AHI	36.8 [23.3, 63.5]
Optimal titration pressure, cm H_2_O	10.0 [9.0, 12.0]
Intentional leak, L/min	26.6 [24.0, 29.2]
Total education time, min	44.8 ± 18.8
PAP optimism	
Extremely optimistic Moderately optimistic Somewhat optimistic	55 (38.2) 55 (38.2) 31 (21.5)
Total Interventions	
0 1 2+	88 (58.7) 32 (21.3) 30 (20)

Values presented as Mean ± SD, Median [P25, P75], or N (column %)

Missing data: PAP optimism (*n* = 144)

### Mask leak and PAP adherence characteristics

**Fig. 1:**
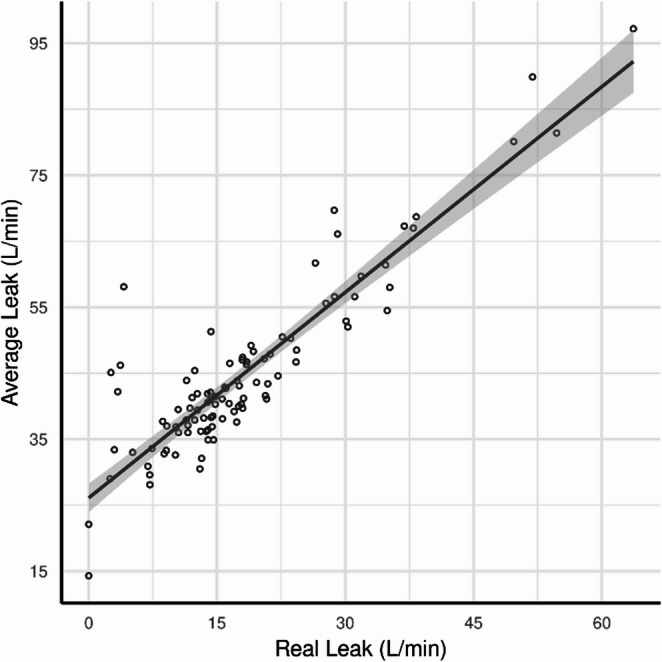
Correlation average leak vs real leak at 3 months. Correlation between average leak and real leak at 3 months. A positive correlation was observed (Pearson’s *r*=0.72, *p*< 0.001). The shaded area represents the 95% confidence interval

After 1 month of PAP usage, only 36.9% of all participants met the adherence criterion with PAP use recorded for ≥ 4 h on 53.3% of days. Median Real Leak was 15.2 L/min [IQR: 11.5, 20.3], and median Average Leak was 42.3 L/min [IQR: 37.7, 48.7]. After 3 months, adherence improved slightly to 46.2%, and the average percentage of days with PAP use ≥ 4 h increased to 63.3%. Leaks improved slightly, with a reduction to 15.5 L/min [IQR: 11.6, 20.8] in Real Leak and 41.4 L/min [IQR: 37.3, 50.2] in Average Leak. Kurtosis of Real Leak was 5.90 compared to 6.19 of Average Leak, indicating that neither variable has extreme leak values contributing to the distribution of their samples. The Skew of Real Leak versus Average Leak was 1.13 to 1.45, indicating that both variables are similarly skewed when compared to a normal distribution and can be considered similar when considering them in correlation and regression analysis. As expected, given its derivation, Real Leak was strongly correlated with Average Leak (Pearson’s *r* = 0.72, *p* < 0.001)), as shown in Fig. 1. Mask leak at the end of month 3 and PAP adherence data are shown in Table 2.

**Table 2:** PAP adherence and leak characteristics at follow-up

1 month	*N*	Statistics
Average PAP use, hr	132	4.1 ± 2.1
Average leak, L/min	132	42.3 [37.7, 48.7]
Real leak, L/min	126	15.2 [11.5, 20.3]
Days ≥ 4 h usage, %	132	53.3 [23.3, 81.3]
Days ≥ 4 h ≥ 70%, %	132	52 (36.9)
3 months		
Average PAP use, hr	132	4.3 ± 2.3
Average leak, L/min	132	41.4 [37.3, 50.2]
Real leak, L/min	126	15.5 [11.6, 20.8]
Days ≥ 4 h usage, %	132	63.3 [31.7, 86.7]
Days ≥ 4 h ≥ 70%, %	132	61 (46.2)

Values presented as mean ± SD, median [P25, P75], or N (column %)

At month 1, adherence was observed in 56.7% of patients using nasal masks vs. 53.3% using nasal pillows and 46.7% using full-face masks, this difference was not statistically significant (*p* = 0.34). At month 3, adherence remained comparable across groups, occurring in 65.0% of nasal mask vs. 58.6% of nasal pillows and 41.7% of full-face mask users (*p* = 0.76).

White race was associated with a higher percentage of days with PAP adherence compared to Black race (63.14 days [55.98, 70.31] vs. 41.33 days [24.38, 58.29], *p* = 0.02). A higher AHI was also significantly associated with greater adherence, with each one-point increase in AHI corresponding to a 0.46% increase in adherence days (*p* < 0.001). Troubleshooting interventions, PAP education time, and PAP optimism did not predict adherence (*p* = 0.30, *p* = 0.46, and *p* = 0.96, respectively).

### Associations between participant characteristics, mask leak, and PAP use/adherence

Associations between mask leak and PAP use/adherence at 3 months are shown in Table 3. Real Leak and Average Leak were highly correlated at 1-month and 3-month timepoints (rho [95%CI]: 1-month 0.73 [0.61,0.81], 3-month 0.90 [0.85,0.93], *p* < 0.001 for both). Both Real Leak and Average Leak had negative correlation with adherence measures at 3 months. For example, when Real Leak increases by 1 L/min, average daily PAP use decreases 2.66 min ([95%CI]: [−4.79, −0.53], *p* = 0.015), and percentage of days with PAP use ≥ 4 h decreases 0.72% ([95%CI]: [−1.24, −0.19], *p* = 0.008). When Average Leak increases by 1 L/min, average daily PAP use decreases by 2.01 min ([95%CI]: [−3.87, −0.15], *p* = 0.035) and percentage of days with PAP use ≥ 4 h decreases by 0.55% ([95%CI]: [−1.01, −0.10], *p* = 0.018). When Real and Average Leak increases by 10 L/min, average daily PAP use decreases by 26.6 and 20.1 min, respectively, and percentage of days with PAP use ≥ 4 h decreases by 7.2% and 5.5%, respectively (*p* = 0.008/0.018). This relationship remains significant both before and after adjusting for age, sex, BMI, race, education, ESS baseline, and AHI.

**Table 3:** Models of average PAP use, adherence and leak variables

Outcome	Primary predictor	Coefficient (95%CI)	*P*-value
Average PAP use (usage per night, minutes)	Real leak 3 months	−2.66 (−4.79, −0.53)	0.01
	Average leak 3 months	−2.01 (−3.87, −0.15)	0.012
PAP adherence (≥ 4 h usage, %)	Real leak 3 months	−0.72 (−1.24, −0.19)	0.001
	Average leak 3 months	−0.55 (−1.01, −0.10)	0.009

Adjusted covariates: age, sex, BMI, race, education, ESS at baseline, AHI

Real Leak was correlated with higher AHI and optimal titration pressure (rho = 0.45, [95%CI]: [0.18,0.72], *p* = 0.001) at 1 month but not 3 months (rho [95%CI]: AHI 0.08 [−0.12,0.27], *p* = 0.45). However, Average Leak was correlated with AHI and optimal titration pressure at both timepoints (1 month: AHI 0.37 [0.18,0.53], *p* < 0.001; 3 months: AHI 0.24 [0.05,0.42], *p* = 0.015).

Real Leak and Average Leak at 1 or 3 months were not associated with the number of troubleshooting interventions (*p* = 0.79; *p* = 0.26 at 3 months), PAP education (*p* = 0.48; *p* = 0.34 at 3 months), or PAP optimism (“Extremely Optimistic vs. somewhat” *p* = 0.10; *p* = 0.81; “Moderately Optimistic vs. somewhat” *p* = 0.89; *p* = 0.31 at 3 months). Marital status was not associated with adherence (married vs. others, *p* = 0.22). Education level was also not associated with adherence when modeled as < college versus ≥ college (*p* = 0.12) or across three categories (≤ high school, > high school and < college, ≥ college; *p* = 0.30).

## Discussion

In a secondary analysis using data from the HomePAP trial involving 139 patients, almost half of which were “4 & 70” PAP therapy compliant, we found: (1) both Average Leak and Real Leak (that eliminates the intentional leak component) were negatively associated with PAP adherence; (2) greater AHI and optimal titration pressure were associated with greater Average and Real Leak at 1 month, but only with Average Leak at 3 months; and (3) troubleshooting interventions, PAP education time, and PAP optimism did not predict adherence at either timepoint. These findings support the AASM’s position on leak as an important factor when choosing a mask for PAP therapy and adds to previous findings that mask leak negatively affects PAP adherence by introducing the concept of Real Leak [11, 14]. 

Although Real Leak and Average Leak demonstrated similar distributions and comparable associations with PAP adherence, Real Leak may still offer conceptual value by explicitly isolating unintentional leak, which could improve clarity and consistency in leak reporting across different mask interfaces, even in the absence of superior predictive performance. Currently, the two most common leak measures are ‘time spent with excessive mask leak’ and ‘amount of leak’ (measured in liters per second over the recording period), with researchers often reporting both [15]. Using the manufacturer’s reported intentional leak per mask/pressure allowed us to derive Real Leak from Average Leak. The device manufacturer (Respironics REMstar Auto) defines Average Time in Large Leak Per Day as the average amount of time spent with excessive leak that will compromise therapy and suggests that large leak may be due to poor mask fitting. In clinical practice, leak ≥ 2x the intentional leak is seen as problematic and warrants troubleshooting in order to optimize adherence and treatment benefits [16]. Even though large leak does not necessarily indicate that the patient is receiving sub-therapeutic pressure, the device’s algorithm will be impacted by the unintentional leak, which may lead to the delivery of suboptimal pressure and less therapeutic effect [17]. Other investigations have shown correlations between leak and PAP adherence using Average Leak or unspecified leak variables, but Real Leak accounts for differences in intentional leak due to different masks or device settings, providing a more clinically meaningful definition [18]. 

Mask leak is often underreported. A recent systematic review found only 21 randomized controlled trials over a 26 year-period reported unintentional leak [9]. Of the papers referenced, multiple leak measurements were used without consistency [19–21]. Even when mask leak is recorded, it is typically grouped with general interface problems (i.e. mask fit) impacting adherence [22]. Mask leak is frequently cited as a barrier to optimal therapy without providing a definition [23]. Even when mask leak is defined, failure to account for intentional leak leads to unreliable associations between leak and adherence [24]. Nonetheless, several studies have identified mask leak as a short term predictor of PAP adherence and observed that interface issues are frequently cited by patients who discontinue PAP [25–27]. Interestingly, while adherence improved modestly between 1 and 3 months, this increase was not explained by PAP education time, troubleshooting interventions, mask interface type, or mask or pressure changes, none of which were independently associated with adherence in multivariable analyses. This pattern likely reflects gradual patient adaptation to PAP therapy rather than the effect of a specific measured intervention.

This study has several limitations. First, follow-up was limited to 3 months, and therefore the long-term impact of Real Leak versus Average Leak on PAP adherence remains unknown. Second, all participants were treated using a single PAP device platform (Respironics REMstar Auto), consequently, the observed associations may not be directly generalizable to devices from other manufacturers that employ different leak definitions or signal-processing algorithms. In addition, participants were recruited from research-intensive academic sleep centers, which may not reflect real-world PAP use, mask availability, staff expertise, or follow-up patterns, thereby limiting generalizability [28]. Third, because Real Leak is derived from Average Leak by subtracting participant-specific intentional leak, these measures share substantial overlapping information, and similar associations with adherence are therefore expected, particularly when variability in intentional leak is modest relative to total leak variability. Finally, mask leak is a dynamic phenomenon, and the use of 30-day averaged leak measures may obscure intra-night and night-to-night variability that could differentially influence comfort, pressure delivery, and adherence.

So, what does leak have to do with it? We explored the relationship between mask leak and adherence in the first randomized controlled trial examining these variables. Although we found that both Real and Average Leak predicted adherence, there was no significant difference between the two measurements. While this raises questions about the clinical utility of distinguishing these measures, current variability in leak nomenclature contributes to confusion and limits practical application. The findings support considering a change in this terminology to better reflect actionable parameters for therapy optimization. PAP devices that enable adjustments for mask type and intentional leak settings offer a potential pathway to address unintentional leak, but their effectiveness depends on consistent use of compatible masks and devices, a practice not yet standardized [29]. Developing clear, clinically relevant cut-points for both Real and Average Leak may provide more actionable insights for therapy optimization. Further research should aim to clarify whether Real Leak provides significant benefits over Average Leak measures in enhancing adherence outcomes long-term.

## Data Availability

The data that support the findings of this study are available from the corresponding author upon reasonable request.

## Abbreviations

AASM: American Academy of Sleep Medicine
AHI: Apnea-hypopnea index
APAP: Auto PAP
BMI: Body mass index
ESS: Epworth Sleepiness Scale
HSAT: Home sleep apnea testing
IQR: Interquartile range
OSA: Obstructive sleep apnea
PAP: Positive airway pressure
PSG: Polysomnography
